# Supplementary material for: Are carbon emissions trading and green financial instruments synergistic? -Comprehensive quantitative research based on content analysis
Source: PLoS One. 2024 Mar 7;19(3):e0298601. doi: 10.1371/journal.pone.0298601 (PMC10919601; doi:10.1371/journal.pone.0298601)
Supplement: S1 Data — (ZIP) [file pone.0298601.s001.zip › Data/地级市Z_F工作报告.zip!/╜¡╦╒/╬▐╬2╩╨/└┤╫╘╣1⁄2╓┌║┼í╛┬φ┐╦╩2╛▌═°í┐_2006.docx]

各位代表：

　　现在，我代表无锡市人民政府向大会报告工作，请予审议，并请市政协委员和其他列席人员提出意见。

2005年工作回顾

　　2005年，全市人民在中共无锡市委领导下，以科学发展观统揽全局，进一步转变发展观念，创新发展模式，提升发展质量，全面完成了市十三届人大三次会议确定的各项任务，在全省率先基本建成全面小康社会。预计实现地区生产总值2805亿元，可比价增长15.1%；财政总收入421.8亿元，同口径增长19%，一般预算收入181.7亿元，同口径增长24.2%；全社会固定资产投资完成1335.1亿元，增长19.8%；城镇登记失业率3.27%；居民消费价格涨幅2%。

　　2005年，市政府重点抓了以下工作：

　　（一）优化产业结构，积极提升经济运行质量

　　推动工业集群发展、高端延伸。国家（无锡）液晶显示产业园等先进制造业基地加快发展，集成电路、液晶显示、生物医药、新材料等产业集群初具规模，博世、TCL、清华同方、柯尼卡美能达等一批项目建成投产，新增国家级高新技术企业19家。30家企业入选全省百强高新技术企业，居全省首位。规模工业增加值完成1353.1亿元，增长22%。营业收入超百亿元企业新增5家，累计达到11家。新增中国名牌产品7个，国家免检产品企业14家。推动服务业规模扩张、层次提升。完成服务业增加值1060亿元，增长14.8%；社会消费品零售总额824.1亿元，同口径增长16.3%。现代服务业快速成长，无锡（国家）工业设计园、无锡（国家）动画产业基地发展态势良好，保税物流园开工建设。大力构建现代市场体系，崇安寺步行街开业，国际招商城、新世界国际纺织服装城等初具规模，新增百亿元大市场2家，累计达到8家。接待入境旅游者65.9万人，旅游总收入301.6亿元。灵山景区服务标准被列为省级标准进行推广。金融态势良好，信贷结构优化，年末各项本外币存贷款余额分别为3343.6亿元和2264亿元，比年初增加579.7亿元和384.8亿元。推动现代农业调整结构、优化布局。阳山万亩水蜜桃、绿羊生态园等5个市级重点园区全面建成，无公害农产品基地扩大到107.3万亩，市级以上农业龙头企业增加到51家，农业适度规模经营集中度43.9%，农机化综合水平88.7%。推动资源节约、要素集约。积极推进循环经济发展，编制实施循环经济发展规划、工业能效指南等政策意见。严格保护耕地，加大复垦开发力度，开发整理新增耕地1.8万亩，建成多层标准厂房1095万平方米。

　　（二）坚持开拓创新，着力增创体制机制优势

　　开放型经济全面拓展。利用外资质量提升，完成到位注册外资（商务部确认口径）20.1亿美元，新批注册资本超亿美元项目3个，新批项目单体规模767.6万美元。服务业协议注册外资15.3亿美元，日本瑞穗实业银行获批落户，外资投资公司、贸易公司和人力资源服务机构首次成功引进。外贸结构转型升级，进出口总额291.9亿美元，增长33.6%，其中出口155.5亿美元，增长41%，高新技术产品出口占比升至40.9%。新批境外投资项目51个，总投资3402.6万美元，项目数保持全省领先。民营经济素质提高。销售超亿元企业达到550家，22家企业进入全国民企五百强，尚德太阳能公司成为中国内地首家在纽约证券交易所上市的民营企业。中小企业担保体系进一步加强，担保公司注册资本金、贷款担保余额继续列全省首位。社会事业改革取得突破。市医院、学校、文化艺术、体育场馆与训练等4个管理中心正式运行，市属义务教育阶段学校实行属地管理，71家行业协会完成'政会脱钩'。投融资体制改革稳步实施。投资渠道拓宽，投资结构改善，外资、民资投入占全部投资的70.1%，服务业投资占全部投资的40.6%。着力扩展基础设施、农业农村、重大项目融资渠道，完善政府投资项目建设管理机制，积极推行项目'代建制'。全市有形建筑市场、土地市场、产权交易市场及部分市政设施建设实行市场化运作。

　　（三）加快城乡建设，切实提高区域现代化水平

　　突出规划龙头作用。不断完善规划体系，推进城乡资源要素集约利用。编制完成市域农村规划、现代服务业规划和轨道交通线网及建设规划等一批重大基础设施规划。加快建成区功能调整和工业企业'退城入园'，城区4个重点片区启动开发，蠡湖新城骨架路网基本形成。强化基础设施建设。五爱路、梁溪路、钱荣路、惠山大道、江阴澄南大道、宜兴教育路及金城公铁立交等重点道桥竣工投用，新建城市道路83公里。312国道和沪宁高速无锡段改拓建完成，芜申运河宜兴绕城段通航。落实房地产市场调控措施，全市竣工经济适用房23万平方米、拆迁安置房541.6万平方米，商品房销售面积398.7万平方米，老新村整治60万平方米。市区整修背街小巷100条，改造供水管网31.2公里，新增天然气用户近11万户，新辟、调整公交线路31条。区域供水工程全面完成，市区日供水能力达到145.6万吨，江阴和宜兴城区分别为70万吨和20万吨。本地网固定电话号码升至八位。不断完善口岸功能。无锡机场新辟航线5条，获准开通香港临时客运包机，全年进出港旅客62万人次，货邮吞吐量增长69.9%。无锡港江阴港区和夏港第二公共型港区建设力度加大，全市港口吞吐量达到5046万吨。加快建设绿色无锡。加强资源保护和环境综合整治，环境质量综合指数达到80.4。空气良好天数占比88.5%，城市水域功能区水质达标率升至59.5%，城市生活污水集中处理率超过70%，省内第一个工业固体废弃物安全填埋场投入运行。大力植树造林，加快惠山青龙山整治，积极推进绿色通道和城市绿地建设，蠡湖及十八湾景区成为全省最大的开放式绿地，宜兴成为国家园林城市，江阴建成国家生态示范区。全市森林覆盖率升至20.3%，城市绿化覆盖率升至40%，人均公共绿地面积增加到10.1平方米。

　　（四）统筹经济社会，有效增强城市综合功能

　　城市影响力有了新提升。入选中国品牌经济城市和福布斯内地最佳商业城市。成功举办第三届太湖博览会和世界粮油大会、世界城市服务业大会。与德国勒沃库森市、意大利维琴察市结为友好城市。科教发展再上新台阶。列入国家'火炬创新试验城市'行列，通过国家技术标准试点城市验收，太湖国际科技园、藕塘职教园加快建设。预计全社会研究与开发费用投入占生产总值比重为1.56%，专利申请量达到5621件。大力发展各类教育，全市高等教育毛入学率达到51.4%，普通高校在校生近8万人。残疾儿童义务教育普及率超过99%，11.5万适龄常住流动人口子女在锡接受义务教育，其中九成以上在公办学校就读。公共管理实现新进步。强化公共卫生职能，市区建成社区卫生服务中心24个，禽流感防治工作取得阶段性成效。落实副食品市场蔬菜残留农药速检措施，药品市场抽验合格率96%。承办'十运会'6大项目办赛任务，乒乓球等3个赛区被评为最佳赛区，无锡籍运动员夺得金牌6枚。继续保持低生育水平，人口自然增长率0.67‰。全面实施农村计划生育家庭奖励扶助制度，发放奖扶金近600万元。文明城市建设迈出新步伐。连续第三次获选全国创建文明城市工作先进市，荣获全国城市畅通工程模范管理城市称号，华西村、官林镇成为全国文明村镇，东港镇成为国家卫生镇，全市注册志愿者人数增加到22万。深入挖掘和保护历史文化遗产，鸿山遗址被列为全国十大考古新发现，无锡成为江苏省历史文化名城。积极打造文化品牌，民族舞剧《红河谷》被评为全国十大舞台精品剧目，市博物馆、钱钟书故居、秦邦宪故居等免费开放。深入开展市容环境专项治理，重点整治改造城乡结合部，加大违法、临时建筑拆除力度，城管创优考评名列全省第一。

　　（五）关注民生民富，促进市民共享发展成果

　　社会就业态势良好。鼓励群众自主创业，推进建立城乡统筹的就业机制，开展农村劳动力转移就业培训。城镇新增就业14.5万人，下岗失业人员再就业1.9万人，本地农村劳动力转移就业近5万人，吸纳苏北地区劳动力9.5万人。居民收入稳步增长。企业工资集体协商制建制面71.8%，企业最低工资标准进一步提高，各项惠农政策全面落实。城市居民人均可支配收入16005元，增长17.8%；农民人均纯收入8004元，增长12.5%；年末居民本外币储蓄存款人均3.2万元，比年初增加近5000元。社会保障统筹推进。国家'金保工程'试点--社会保障卡工程主体项目基本建成。全市企业养老、医疗、失业、工伤、生育等保险参保人数分别增加19.2万、23.4万、7.6万、14.8万和12.5万，城镇养老、医疗、失业等保险综合覆盖率均超过96%。新型农民养老保险参保人数43.8万，覆盖面72%，各级财政对新型农村合作医疗的筹资额达到人均73元，被征地农民基本生活保障率100%。城乡低保对象应保尽保，共发放低保金7900多万元、各项慈善救助金1600多万元。实事项目全面完成。主城区开工建设公共停车场4个。向694户低保家庭提供廉租房。2006年元旦起，70岁以上老人可持公交卡免费乘坐市区75条线路的公交车。为100名贫困残疾人免费安装义肢，为400名贫困精神病患者提供免费药物，为40名残疾孤儿实施康复手术。百村奔小康工程成效显现，新增脱贫转化村39个，完成农村公路建设483公里，各镇自来水入户率95%，农村卫生户厕普及率86.4%。平安无锡创建深入开展。全力推进'大防控'，加大严打整治力度，严惩'搬霸'等违法犯罪行为，我市成为全省首批'社会治安安全市'。巩固提高'大调解'，进一步做好信访工作，及时妥善处理各类矛盾。加大安全隐患整治和事故查处力度，事故起数和死亡人数实现'双下降'。

　　（六）转变政府职能，努力提高行政执行能力

　　严格依法行政。贯彻执行市人大及其常委会的各项决议，扎实办理人大代表的建议、批评、意见和政协委员的提案及建议案。进一步加强政府法制工作，保持规章、规范性文件的合法性、严肃性。修订完善了《无锡市人民政府工作规则》，进一步健全集体领导和集体决策制度，完善决策程序和规则。深化行政审批制度改革，全面清理行政许可事项，审批事项再削减31 %，审批集中度提高到81.7%。完善管理机制。提高政府快速反应能力，妥善处理各类突发公共事件。健全价格调控体系，稳定市场物价水平。认真履行统计职能，完成全国经济普查工作任务。完善预算管理制度，深化非税收入改革，全面推行市级国库集中支付，扩大政府采购范围。进一步优化支出结构，市本级财政支农支出增长25.6%，科技支出增长19.3%，教育支出增长22%。提高行政效能。推进'阳光政府'建设，成立市效能投诉中心，开展机关效能监察。推广电子政务，公文传输实现无纸化。建立政府新闻发言人制度，完善市长公开电话和市长信箱工作机制，畅通民声民情反映渠道，努力帮助基层和群众解决实际困难。全面履行职责。审计、外事、侨务、对台、接待、人事、人武、人防、双拥、民族宗教、气象、防震减灾等工作取得新成绩，史志、档案、广电、新闻出版、妇女、儿童、老龄、残疾人等事业有了新发展。

　　各位代表，2005年无锡率先基本建成全面小康社会所取得的成绩，是全体无锡市民务实奋进、开拓创新的结果。这里，我代表无锡市人民政府，向辛勤工作、积极奉献的无锡市民，向忠实履行职责的各界人士，向中央及省在锡部门，向驻锡人民解放军和武警官兵、全市公安干警，向在锡的外来建设者和所有关心支持无锡发展的海内外友人，表示衷心感谢并致以崇高敬意！

　　回顾去年工作，我市经济社会发展取得了令人鼓舞的成绩，但对照科学发展观的要求，促进经济社会全面协调可持续发展任务依然艰巨、工作依然繁重。人口、资源、环境矛盾仍然突出，促进可持续发展的要求更加迫切；城乡之间、经济社会之间发展不够协调的问题仍较明显，全面推进统筹发展的要求更加迫切；现代服务业发展相对滞后，先进制造业整体竞争力有待提高，加快产业结构优化升级的要求更加迫切；自主创新能力不强，企业的创新主体地位还未能完全确立，完善创新机制的要求更加迫切；群众对医疗、教育、住房等问题反映较多，社会保障体系还不够完善，加强社会管理和公共服务的要求更加迫切；影响城市安全的因素增加，因利益调整而引发的社会矛盾时有发生，维护社会和谐的要求更加迫切。同时，政府工作中还存在着不足和薄弱环节，政府职能转变和自身建设还需要不懈努力。这些都要求政府进一步增强责任意识，不断提升工作的预见性、开创性和主动性，努力把各项工作做得更好。

　　 '十五'主要成就和'十一五'奋斗目标

　　刚刚过去的'十五'时期，是我市发展史上综合实力提升很快、城乡面貌变化巨大、人民群众得到实惠较多的黄金发展期之一。这五年，我们以率先发展统揽全局，全面推进区域现代化。按照争创全省'两个率先'先导区示范区的目标定位，努力推动经济发展进入新一轮快速上升期，经济运行稳定性明显提高，经济发展协同性不断改善，地区生产总值年均增长14.5%左右，五年翻番有余，实现了争先进位。财政总收入五年增长3.7倍多，年均增长31.3 %，占地区生产总值比重由'九五'末的9.3 %提高到'十五'末的15%，发展质量和效益显著提升。更加注重推进社会发展，不断加大社会事业投入，集中建设了一批社会事业项目和公益性设施，科教文卫体等各项事业发展活力增强、水平明显提升，社会发展指数跃居并保持全省第一。这五年，我们以对外开放为主线，全面推进经济国际化。坚持'引进来'与'走出去'相结合，不断提高利用外资水平，积极放大开放园区示范带动效应，有效发挥外贸拉动作用，开放型经济对经济社会发展的促进作用日益显现，成为无锡经济发展新优势。五年共计批准外资项目3800多个，累计引进超亿美元项目48个，到位注册外资超过115亿美元，出口总额突破420亿美元。这五年，我们以转变增长方式为着力点，全面推进产业高度化。实施制造业和服务业'双轮驱动'战略，积极推进产业升级，推进布局集中、用地集约、产业集聚，推进技术创新与进步，打造国际先进制造业基地和区域性服务业高地。工业经济综合实力保持领先，高新产业增加值占规模工业比重升至33.4%，比'九五'期末提高13.4个百分点。商贸物流业、旅游业、房地产业等发展迅猛，消费升级趋势明显，增长动力结构日益优化。这五年，我们以改革创新为动力，全面推进经济体制市场化。以国有企业产权制度改革为中心环节，推进各项配套改革，加快转变政府职能，初步形成与市场经济要求相适应的微观主体基础与经济运行机制。国有企业改革任务基本完成，国有资产管理体制基本建立。大力激发民间创业潜能，不断推动民营经济快发展、上规模、提素质，使民营经济成为增量经济、富民经济、支柱经济。民营经济占全市经济的比重上升到51.2%。这五年，我们以统筹协调为基本原则，全面推进城乡一体化。以完善城乡布局为基础，以基础设施、公共设施建设为抓手，以推进城乡制度接轨为重点，突出完善市域城市体系，突出实施'城市南进、工业北移'的中心城市发展战略，突出改善农村生产生活条件，突出构筑人与自然和谐的生态环境。市域三级城市体系规划确定、形态初现，城市化率预计达到67%。这五年，我们以安民富民为立足点，全面推进群众生活宽裕化。从促进就业、鼓励创业入手，强化居民收入稳定增长机制，强化社会保障体系建设，强化社会救助措施，促进城乡居民共同迈进小康社会。城市居民人均可支配收入和农民人均纯收入年均分别增长13.2%和8.8%，五年新增城镇就业岗位60万个，农村劳动力转移就业22万多人。

　　进入'十一五'，我们面临着机遇与挑战并存、机遇大于挑战的发展环境。随着人民币汇率变化出现新情况，国际能源市场变化带来新压力，外贸出口环境变化形成新挑战，经济运行中的不稳定不确定因素趋于增多。我国入世过渡期结束，围绕资源、市场、技术、人才等要素的国际竞争将更加激烈，无锡将日益感受到来自国内外的竞争压力。然而应该看到，经济全球化加快推进的趋势没有改变，世界经济结构调整和产业转移的走势没有改变，我国经济保持平稳较快增长的态势没有改变，无锡实现持续快速发展具备很多有利条件。近年来，区域经济合作密切，将为下一轮发展提供新的机遇；经济结构调整和城市化进程加快，将为提升发展水平开辟新的空间；社会主义市场经济体制进一步完善，将为加快发展注入新的活力；工业化进程加速、产业升级提速、民营经济和现代服务业发展增速，将为率先发展增添新的动力。只要我们充分利用国内外各种有利条件，创新思路，科学谋划，扎实工作，未来五年的发展完全可以大有作为。

　　根据《中共无锡市委关于制定无锡市国民经济和社会发展第十一个五年规划的建议》，紧紧围绕争创全省'两个率先'先导区示范区总目标和全面落实科学发展观总要求，突出社会主义市场经济条件下发展规划的宏观性、战略性、政策性和指导性，市政府组织编制了《无锡市国民经济和社会发展第十一个五年规划纲要（草案）》（以下简称《纲要》），提请大会审议。

　　《纲要》提出：'十一五'乃至以后一个时期，无锡发展的基本定位是由全面小康向基本实现现代化目标迈进，加快建设国际先进制造技术中心和区域性商贸物流中心、创意设计中心、职业教育中心、旅游度假中心，努力把无锡打造成为最适宜投资创业的工商名城、最适宜创新创造的设计名城、最适宜生活居住的山水名城、最适宜旅游度假的休闲名城、最富有人文特质的文化名城。到2010年，确保在省内率先基本实现现代化，确保经济社会转入全面协调可持续发展轨道，把无锡建设成为经济繁荣、生活富裕、科教发达、环境优美、法制健全、社会文明的和谐宜人新无锡。主要发展目标是：地区生产总值在2005年基础上实现翻番，人均地区生产总值预期超过1万美元（按照常住人口测算）；主要污染物排放量和万元地区生产总值能耗分别比'十五'期末降低20%；森林覆盖率和城市绿化覆盖率分别达到27%和45%，环境综合指数达到90，力争建成国家生态城市群；全社会研发费用投入占生产总值比重达到2.5%；城市居民人均可支配收入和农民人均纯收入年均增长10%以上。

　　根据以上基本定位和总体目标，'十一五'期间必须进一步明晰战略思路，牢牢把握工作主动权。坚持又快又好发展，是'十一五'发展的主题。始终不渝地加快发展、率先发展，这一条任何时候任何情况下决不能有任何动摇。我们所要的率先发展，是立足于快、着眼于好的率先发展。快，就是经济发展比全国、全省平均速度快，努力保持经济总量在全国大中城市中的领先地位；好，就是要切实转变经济增长方式，加快科技进步、体制创新，着力推进节约和集约发展，不断提高经济增长的质量和效益。加快富民步伐，是'十一五'发展的出发点和归宿点。实现好最广大人民群众的根本利益，是落实科学发展观的根本要求，也是经济工作的首要任务。必须坚持富民优先不动摇，以更大力度、更实举措，积极鼓励民众创业致富，努力扩大城乡就业，千方百计增加居民收入，加快完善社会保障体系，不断提高人民生活质量，让群众真正过上富裕生活。深化改革、扩大开放，是'十一五'发展的动力源泉。必须坚持以解放思想为先导，进一步完善所有制结构，建立健全现代市场体系，深入推进政府管理体制改革，为科学发展提供体制保障。必须坚持全方位扩大开放，在更广领域、更高层次上参与国际经济合作与竞争，全面提高对外开放水平和城市国际化程度，实现市场化、国际化与工业化、城市化、信息化的互动并进。建设创新型城市，是'十一五'发展的战略基点。必须从增强城市创新能力和解决现实紧迫需要出发，加快建立以企业为主体、市场为导向、产学研结合的技术创新体系，加强原始创新、集成创新和引进消化吸收再创新，大力开发具有自主知识产权的关键技术、核心技术和知名品牌，促进产业升级，推动'无锡制造'向'无锡创造'提升，支撑经济社会持续快速发展。强化统筹协调，是'十一五'发展的重要方针。必须更加注重统筹兼顾，采取有力措施，加强薄弱环节，突破难点问题，着力形成统筹发展的有效机制。进一步提高经济社会协调发展水平，强调经济发展与人口、资源、环境相协调，努力建设资源节约型、环境友好型社会。探索城乡统筹发展路子，推进社会主义现代化新城乡建设，提升区域城市化水平，积极参与以上海为龙头的长三角经济一体化进程。构建和谐社会，是'十一五'发展的基本任务。必须按照以人为本原则，把构建和谐社会纳入'两个率先'目标内涵，认真解决人民群众最关心、最直接、最现实的利益问题，妥善处理各方面利益关系，维护和促进社会公平。进一步加强精神文明建设和民主法制建设，充分激发全社会的创造活力，促进社会全面进步和人的全面发展。
